# Rxn-INSIGHT: fast chemical reaction analysis using bond-electron matrices

**DOI:** 10.1186/s13321-024-00834-z

**Published:** 2024-03-29

**Authors:** Maarten R. Dobbelaere, István Lengyel, Christian V. Stevens, Kevin M. Van Geem

**Affiliations:** 1 Laboratory for Chemical Technology, Department of Materials, Textiles and Chemical Engineering, Faculty of Engineering and Architecture, Ghent University, Technologiepark 125, 9052 Ghent, Belgium; 2 ChemInsights LLC, Dover, DE 19901 USA; 3 SynBioC Research Group, Department of Green Chemistry and Technology, Faculty of Bioscience Engineering, Ghent University, Coupure Links 653, 9000 Ghent, Belgium

**Keywords:** Cheminformatics, Chemical reactions, Reaction analysis, Reaction classification, Reaction conditions

## Abstract

The challenge of devising pathways for organic synthesis remains a central issue in the field of medicinal chemistry. Over the span of six decades, computer-aided synthesis planning has given rise to a plethora of potent tools for formulating synthetic routes. Nevertheless, a significant expert task still looms: determining the appropriate solvent, catalyst, and reagents when provided with a set of reactants to achieve and optimize the desired product for a specific step in the synthesis process. Typically, chemists identify key functional groups and rings that exert crucial influences at the reaction center, classify reactions into categories, and may assign them names. This research introduces Rxn-INSIGHT, an open-source algorithm based on the bond-electron matrix approach, with the purpose of automating this endeavor. Rxn-INSIGHT not only streamlines the process but also facilitates extensive querying of reaction databases, effectively replicating the thought processes of an organic chemist. The core functions of the algorithm encompass the classification and naming of reactions, extraction of functional groups, rings, and scaffolds from the involved chemical entities. The provision of reaction condition recommendations based on the similarity and prevalence of reactions eventually arises as a side application. The performance of our rule-based model has been rigorously assessed against a carefully curated benchmark dataset, exhibiting an accuracy rate exceeding 90% in reaction classification and surpassing 95% in reaction naming. Notably, it has been discerned that a pivotal factor in selecting analogous reactions lies in the analysis of ring structures participating in the reactions. An examination of ring structures within the USPTO chemical reaction database reveals that with just 35 unique rings, a remarkable 75% of all rings found in nearly 1 million products can be encompassed. Furthermore, Rxn-INSIGHT is proficient in suggesting appropriate choices for solvents, catalysts, and reagents in entirely novel reactions, all within the span of a second, utilizing nothing more than an everyday laptop.

**Supplementary Information:**

The online version contains supplementary material available at 10.1186/s13321-024-00834-z.

## Introduction

The design of entirely new synthesis routes remains one of the key challenges in medicinal chemistry [1]. For over half a century, computer tools have been developed to assist synthetic organic chemists in this task [2–5], but it was the arrival of powerful computing resources, complex algorithms and large databases in chemical sciences and engineering that has tremendously accelerated the applicability of computer-aided synthesis planning (CASP) tools [6–8]. A first example is the development of retrosynthetic software, which breaks down a molecule into simpler precursor compounds, using chemical knowledge from millions of reactions [9–12]. The first retrosynthesis tools date back to the 1960s and relied on the principle of tree search [13]. Today’s computers are not as limited in core memory anymore and programming is far more straightforward, but still the algorithms are based on the same principle of searching a synthesis tree [11]. Another major research topic is designing reactions in forward direction by predicting the reaction outcome [5]. Forward reactivity prediction tasks, in which the product is predicted given the precursors, have also benefited from data availability and machine learning algorithms in tools with satisfying accuracy [14–18]. The third major task in CASP is reaction condition prediction, but this is a missing link in automated synthesis platforms as it still requires the intervention of human chemists [19].

Automated reaction condition prediction can be thought of as optimizing the reaction conditions or as providing an initial guess that researchers can start from. Usually, optimization is performed for one specific reaction of a given type and the optimization is performed using active machine learning algorithms, such as Bayesian optimization, in which new experiments are queried iteratively for the specific target reaction of the same type [20, 21]. These active machine learning models are able to find better reaction conditions that have not been reported before in literature. Other machine learning models such as neural networks have been applied for reaction condition prediction without the need for new experiments, but they tend to capture literature trends rather than proposing new and better conditions [22]. Hence, direct prediction models are more suited as initial condition guessers which can be trained on data of one reaction type [23] or on very large, diverse datasets [24]. Furthermore, machine learning models for reaction condition prediction are limited by the accuracy and reproducibility of the reported data in reaction databases [25]. So far, only a fraction of the chemical literature is publicly available thanks to the screening of US patent data [26] and the Open Reaction Database initiative [27].

Despite the availability of many advanced tools, most chemists look for new reaction conditions by screening the scientific literature and comparing the new reaction with similar, known examples. For this similarity matching, we can look at the type of reaction, the involved functional groups, and the scaffold of the molecule. Carey et al*.* [28] defined twelve categories to classify all reactions: heteroatom alkylation and arylation, acylation, aromatic heterocycle formation, C–C bond formation, protection, deprotection, functional group interconversion, functional group addition, oxidation, reduction, resolution and miscellaneous. These categories have been adopted in future studies for analyzing chemical reaction data [29–31]. Schneider et al*.* [30] used commercial software to classify reactions into more specific reaction motifs, such as “Williamson ether synthesis” or “Nitration”, by defining SMIRKS [32], a string-based notation for reaction transformations, for each reaction motif [9]. Only small sets of expert-defined SMIRKS patterns have been made publicly available so far, such as 58 reaction rules from Hartenfeller et al*.* [33] and more than 100 SMIRKS from Avramova et al*.* [34]. Reaction transformations can also be extracted automatically as reaction templates [35, 36] but, in contrast to the reaction rules, many templates might exist for one specific reaction, depending on the functional groups and rings involved in the reaction center. The number of ring systems and functional groups in bioactive molecules is limited, as shown by Visini et al*.* [37], who discovered that there are more than 900,000 potential ring systems with up to 4 fused rings and 14 atoms. However, less than 40,000 ring systems are reported so far [38]. This is in line with what holds for functional groups, where Ertl found that of 3080 functional groups in bioactive molecules, just 768 occur in more than 10 molecules [39]. More than 150 million chemical reactions are out in the scientific literature [40] and it thus appears that these reactions can all be classified with a relatively concise set of reaction classes, reaction names, chemical rings, and functional groups.

In this work we report Rxn-INSIGHT, an open-source framework for efficient analysis of chemical reactions using Reaction SMILES identifiers [32] as sole input. A description of the workflow and the details of the algorithm are given in Sect. “Algorithm”. In Sect. “Results and Discussion”, we discuss the major applications of Rxn-INSIGHT using 1.8 million reactions from the USPTO patent database [26]. The first application consists of classifying and naming the reactions. In the second application, functional groups, rings, and molecular scaffolds are extracted from the products and the reaction centers. Finally, it is illustrated as a side application how Rxn-INSIGHT can be used to suggest initial reaction conditions with the Heck reaction as case study.

## Algorithm

### Overview

**Fig. 1:**
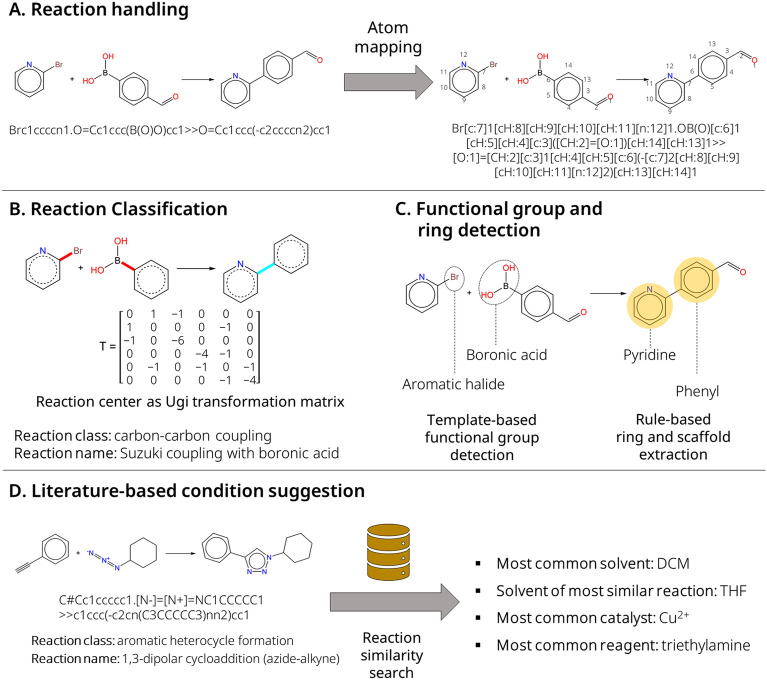
Overview of the algorithm’s workflow. **A** Example of a chemical reaction and its Reaction SMILES with and without atom mapping. **B** Ugi transformation matrix of an example reaction with classification. **C** Extraction of functional groups, rings, and scaffolds from reactants and products. **D** Suggestion of reaction conditions based on the reaction class, scaffold, and functional groups

Rxn-INSIGHT starts from a rule-based algorithm and is designed to efficiently handle chemical reaction data and suggest reaction conditions. It makes extensive use of RDKit [41], one of the most widely used cheminformatics frameworks. Figure 1 illustrates the algorithm’s workflow. The Reaction SMILES identifier of a chemical reaction is the only required input. The reaction does not even have to be balanced. As depicted in Fig. 1a, the chemical reaction is provided with atom mapping to make correspondence between the atoms in the reactants and the products. RXNMapper from Schwaller et al*.* [42] is used for the atom mapping tasks, as it was found to outperform other tools in an independent study [43]. The Rxn-INSIGHT algorithm has three main features: reaction classification using bond-electron matrices and reaction naming (Fig. 1b), detection of functional groups and rings (Fig. 1c), and suggestion of reaction conditions (Fig. 1d). The code is available as open-source software on https://github.com/mrodobbe/Rxn-INSIGHT.

### Bond-electron matrices

**Scheme 1:**
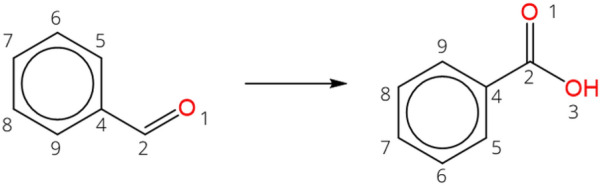
Atom-mapped oxidation reaction from benzaldehyde to benzoic acid

Chemical reactions are classified into reaction classes by a bond-electron (BE) matrix of the reaction center. The concept of BE matrices is introduced by Dugundji and Ugi in 1973 [44], as an extension of Spialter’s atom-connectivity matrix [45]. Reactants and products of a sanitized reaction are represented by an $N\times N$ nonnegative, symmetric BE matrix, with $N$ being the number of heavy atoms in the system. Note that many reactions from chemical databases are not balanced and that, therefore, the number of atoms in the reactants does not necessarily have to be equal to the number of atoms in the products. The unbalanced oxidation reaction from benzaldehyde to benzoic acid (Scheme 1) is taken as example to illustrate the BE matrix concept. Equation (1) gives the $9\times 9$ matrices ${M}_{R}$ and ${M}_{P}$ of respectively the reactant benzaldehyde and the product benzoic acid.

1 $${M}_{R}= \left[\begin{array}{c}\begin{array}{ccccccccc}4& 2& 0& 0& 0& 0& 0& 0& 0\\ 2& 0& 1& 0& 0& 0& 0& 0& 0\\ 0& 1& 0& 1.5& 0& 0& 0& 1.5& 0\\ 0& 0& 1.5& 0& 1.5& 0& 0& 0& 0\\ 0& 0& 0& 1.5& 0& 1.5& 0& 0& 0\\ 0& 0& 0& 0& 1.5& 0& 1.5& 0& 0\\ 0& 0& 0& 0& 0& 1.5& 0& 1.5& 0\\ 0& 0& 1.5& 0& 0& 0& 1.5& 0& 0\\ 0& 0& 0& 0& 0& 0& 0& 0& 0\end{array}\end{array}\right]\begin{array}{c}\mathrm{O }1\\ \mathrm{C }2\\ \mathrm{C }4\\ \mathrm{C }5\\ \mathrm{C }6\\ \mathrm{C }7\\ \mathrm{C }8\\ \mathrm{C }9\\ \mathrm{O }3\end{array}\, {M}_{P}= \left[\begin{array}{c}\begin{array}{ccccccccc}4& 2& 0& 0& 0& 0& 0& 0& 0\\ 2& 0& 1& 0& 0& 0& 0& 0& 1\\ 0& 1& 0& 1.5& 0& 0& 0& 1.5& 0\\ 0& 0& 1.5& 0& 1.5& 0& 0& 0& 0\\ 0& 0& 0& 1.5& 0& 1.5& 0& 0& 0\\ 0& 0& 0& 0& 1.5& 0& 1.5& 0& 0\\ 0& 0& 0& 0& 0& 1.5& 0& 1.5& 0\\ 0& 0& 1.5& 0& 0& 0& 1.5& 0& 0\\ 0& 1& 0& 0& 0& 0& 0& 0& 4\end{array}\end{array}\right]\begin{array}{c}\mathrm{O }1\\ \mathrm{C }2\\ \mathrm{C }4\\ \mathrm{C }5\\ \mathrm{C }6\\ \mathrm{C }7\\ \mathrm{C }8\\ \mathrm{C }9\\ \mathrm{O }3\end{array}$$

The BE matrix $M$ of a molecule has the number of free unshared valence electrons on the diagonal values ${M}_{ii}$. The off-diagonal entries ${M}_{ij} (i\ne j)$ indicate the bond order between two atoms $i$ and $j$. The bond order is defined as follows: ${M}_{ij}=0$ if there is no bond, ${M}_{ij}=1$ for a single bond, ${M}_{ij}=1.5$ for an aromatic bond, ${M}_{ij}=2$ for a double bond, ${M}_{ij}=3$ for a triple bond. The reaction is represented by the $R$-matrix, which is defined as the difference between the BE-matrices of the products and the BE-matrices of the reactants. The R-matrix for the oxidation reaction from Scheme 1 is given in Eq. (2).

2 $$R={M}_{P}-{M}_{R}= \left[\begin{array}{c}\begin{array}{ccccccccc}0& 0& 0& 0& 0& 0& 0& 0& 0\\ 0& 0& 0& 0& 0& 0& 0& 0& 1\\ 0& 0& 0& 0& 0& 0& 0& 0& 0\\ 0& 0& 0& 0& 0& 0& 0& 0& 0\\ 0& 0& 0& 0& 0& 0& 0& 0& 0\\ 0& 0& 0& 0& 0& 0& 0& 0& 0\\ 0& 0& 0& 0& 0& 0& 0& 0& 0\\ 0& 0& 0& 0& 0& 0& 0& 0& 0\\ 0& 1& 0& 0& 0& 0& 0& 0& 4\end{array}\end{array}\right]\begin{array}{c}\mathrm{O }1\\ \mathrm{C }2\\ \mathrm{C }4\\ \mathrm{C }5\\ \mathrm{C }6\\ \mathrm{C }7\\ \mathrm{C }8\\ \mathrm{C }9\\ \mathrm{O }3\end{array}$$

By this definition, the R-matrix is a representation of the reaction center, as the off-diagonal values indicate increase or decrease in bond order and the diagonal values indicate the gain or loss of valence electrons. For practical use, the $R$-matrix is transformed into transformation matrix $T$ in which all zero rows and columns are stripped from the $R$-matrix. The $T$-matrix for the example reaction is given in Eq. (3).

3 $$T= \left[\begin{array}{c}\begin{array}{cc}0& 1\\ 1& 4\end{array}\end{array}\right]\begin{array}{c}\mathrm{C }2\\ \mathrm{O }3\end{array}$$

From the $T$-matrix it is understood which heavy atoms are involved in the reaction center. In this example, a new bond is formed between the carbon atom with map number 2 and the oxygen with map number 3. The value 4 in Eq. (3) indicates the addition of an atom with four free, unshared valence electrons that was not present in the reactants.

### Reaction classification and naming

Reactions are classified into classes that reflect the chemical transformation that happens but not the equipment that is used. The ten classes that were suggested by Carey et al*.* [28] are used: acylation, aromatic heterocycle formation, C–C coupling, heteroatom alkylation and arylation, functional group addition, functional group interconversion, protection, deprotection, oxidation, and reduction. Reactions that do not match any of the criteria are classified as “Miscellaneous”. Enantiomers cannot be distinguished with the BE-matrix approach and are for this reason not considered in this work. Any resolution is, hence, classified as a miscellaneous reaction. Because the reaction categories are rather broad, there are reactions that can be classified into multiple classes. For example, methylation reactions are an example that can be classified as a functional group addition, a C–C coupling, a heteroatom alkylation or a protection. Another example is the reaction between an amine and the tert-butyloxycarbonyl (Boc) protecting group. This reaction should be classified as a protection, but since a carbamate is formed from an amine and anhydride, the reaction could technically also be classified as an acylation reaction.

A reaction is evaluated against a sequence of ten functions, that each represent one of the aforementioned reaction classes. The evaluation process proceeds in a specific order and as soon as a matching reaction class is found, the loop is terminated. The reaction is then assigned to the first class it matched. Table 1 lists the ten reaction classes in the order of evaluation, together with the general rule that the evaluation function is based on. The first class in the loop is that of the aromatic heterocycle formations, which are characterized by a + 0.5 or + 1.5 change in the $T$ matrix. This transformation indicates the formation of an aromatic bond, which is not observed in any other reaction class.

**Table 1:** Classification order of different reaction classes and the general rule used in the classification scheme

Order	Reaction class	General rule
1	Aromatic heterocycle formation	+ 0.5 or + 1.5 values in $T$-matrix Formed ring contains at least one heteroatom
2	Acylation	+ 1 in $T$ matrix corresponds to a bond formed between a heteroatom and the acyl carbon
3	Functional group interconversion	A column in the $T$ matrix has sum zero and contains only + 1 and − 1
4	Reduction	Nonpositive $T$ matrix, nonzero elements on diagonal correspond to oxygen atoms
5	Oxidation	Nonnegative $T$ matrix, nonzero elements on diagonal correspond to oxygen atoms
6	Functional group addition	Nonnegative $T$ matrix, diagonal contains nonzero elements corresponding to heteroatoms
7	Carbon–carbon coupling	+ 1 in $T$ matrix corresponds to a new bond formed between two carbon atoms
8	Heteroatom alkylation & arylation	+ 1 in $T$ matrix corresponds to a new bond formed between a heteroatom and non-acyl carbon
9	Protection	+ 1 in $T$ matrix corresponds to a new bond formed between a heteroatom or alkyne carbon and a carbon or silicon
10	Deprotection	Nonpositive $T$ matrix, − 1 in $T$ matrix corresponds to a broken bond between a heteroatom or alkyne carbon and a carbon or silicon

The classification rules of several reaction classes resemble each other, so that the order in Table 1 is chosen to avoid misclassifications as much as possible. For example, the constructive reaction classes (acylation, heteroatom alkylation and arylation, carbon–carbon coupling) and the protections are all characterized by a $+1$ in the $T$-matrix, which indicates an increase in bond order. To match the reaction class, the bond order increase must correspond to a new bond formed between two atom types. In acylations, this is a new bond between a heteroatom (N, O, S) and a carbon that is part of an acyl group. An exception is made for carbamate formations, which is a common amine protecting group. In case a carbamate is formed, there will not be a match with the acylation function. Heteroatom alkylations and arylations are defined by a new bond formation between a heteroatom (N, O, S) and a carbon, which is not part of an acyl group. In carbon–carbon couplings, there must be a $+1$ in the $T$-matrix that refers to a newly formed carbon–carbon bond. Despite the name, Friedel–Crafts acylations are classified as carbon–carbon couplings (see also Roughley et al*.* [29]) because there are no heteroatoms involved.

Functional group interconversions (FGI), reductions, oxidations, and functional group additions (FGA) are all modifying reactions. To these reactions, a carbon balance constraint is imposed, since during the course of these reactions the number of carbons should remain constant. Exceptions are added to include some carbon-containing functional groups, such as tosyl groups. FGIs are in general distinguished by the presence of a $T$-matrix column that sums up to zero and contains opposing elements. This pattern means that a bond is broken, and a new bond is immediately formed with the same atom. Reductions and deprotections are very similar in terms of $T$-matrices, since both reaction classes have a nonpositive $T$ matrix. This indicates that there are only bond order reductions or reductions in number of free valence electrons. For deprotections, the bond order reductions correspond to broken bonds between an atom in a functional group and an atom in a protecting group. A protection or deprotection is detected when either a heteroatomic functional group or an alkyne is involved in the reaction center. Opposite to reductive reaction classes, are oxidations and FGAs which have a nonnegative $T$ matrix. The positive off-diagonal values indicate increases in bond orders. In oxidations, the positive diagonal values only refer to oxygen atoms, while in FGAs they can be any heteroatom.

To all reaction classes, exceptions are added to enhance classification accuracy. The complete classification scheme is available in the “classification.py” file in the GitHub repository. If a reaction does not match any of the requirements, it is classified as a miscellaneous reaction.

Reaction naming is performed by matching the Reaction SMILES with SMIRKS motifs in RDKit [32, 41]. A set of 527 named SMIRKS reaction motifs is constructed and manually curated. This list is an extension of the 58 SMIRKS from Hartenfeller et al. [33] and is found in Additional file 2.

### Detection of functional groups, rings, and scaffolds

**Fig. 2:**
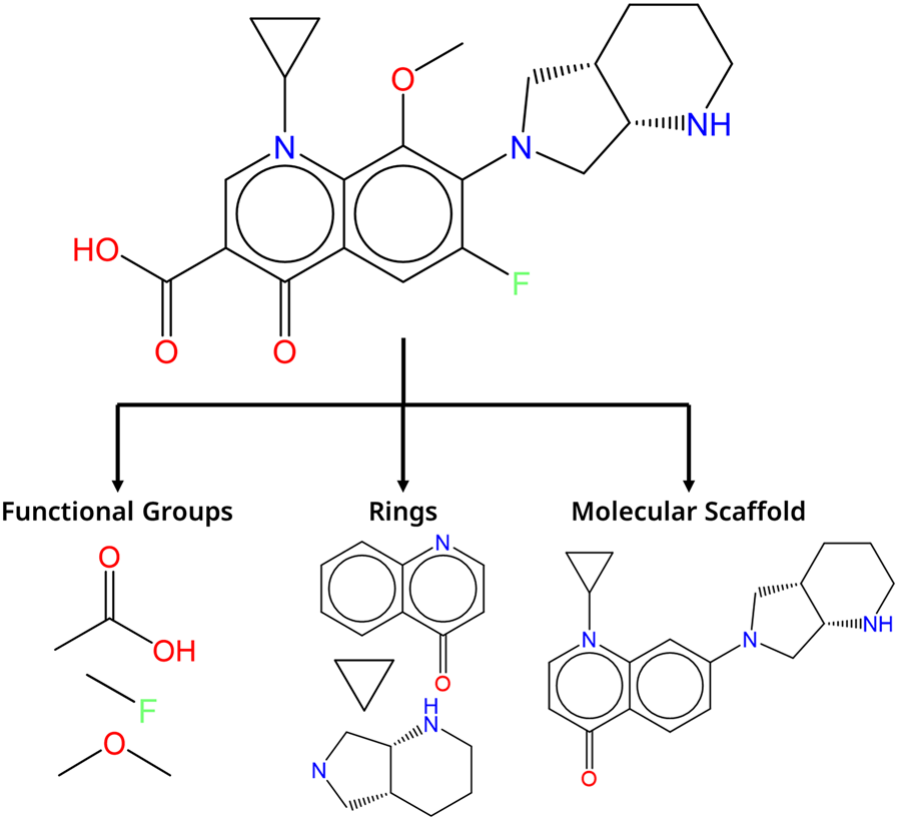
Fragmentation of a molecular structure into functional groups, ring structures, and a Bemis-Murcko molecular scaffold

Figure 2 illustrates the fragmentation of a molecule into functional groups, rings, and a molecular scaffold. A list of 107 functional groups in SMARTS [46] notation is constructed from Ertl’s list of most common functional groups [39]. Detection of functional groups is performed with the substructure matching function in RDKit [41] and each atom can only be in one functional group. If the atom matches two functional groups, the largest group is selected. A functional group is said to participate in the reaction center if at least one of the atoms is represented in transformation matrix $T$.

Ring systems are detected using the RDKit method for ring system counting [41]. The ring is then cleaned up by removing any substituents, with the exception of non-halogen atoms that are only connected to a ring atom (not considering bonds with hydrogen atoms), e.g., phenol or a carbonyl oxygen on an aliphatic ring. A double bond that connects a ring carbon with a non-ring atom is also considered to be part of the ring system as a conjugated ring. After removal of the substituents, hydrogen atoms are added where needed to satisfy regular valences, so that the individual ring system atoms are all closed-shell atoms. The rings are identified by the canonical SMILES of the ring system. In case an atom is part of multiple ring systems (e.g. fused rings), then the largest system is considered.

The reaction center is obtained in SMIRKS using RDChiral, a wrapper around the open-source cheminformatics software RDKit [36]. RDChiral is slightly modified in Rxn-INSIGHT’s implementation so that users can now choose the radius of atoms around the reaction center to be included in the reaction center. Since SMIRKS are not valid reaction SMILES, the reaction center is updated to obtain valid reaction SMILES. The main update concerns ring atoms, because rings can be incomplete in SMIRKS notation but this would raise a semantic error in SMILES. Hence, the complete ring system is included in the reaction SMILES of the reaction center, if at least one atom of that ring is participating in the reaction center or if a functional group that is directly attached to the ring participates in the ring.

The molecular scaffold is the core of a molecule to which functional groups are connected. In medicinal chemistry, scaffolds are a vital concept in the design of new bioactive compounds. RDKit is used to extract the Bemis-Murcko (BM) scaffold from the main reaction product [47]. The BM scaffold is created by removing all side chains from a molecular structure so that only ring and linker atoms remain.

### Reaction similarity

**Fig. 3:**
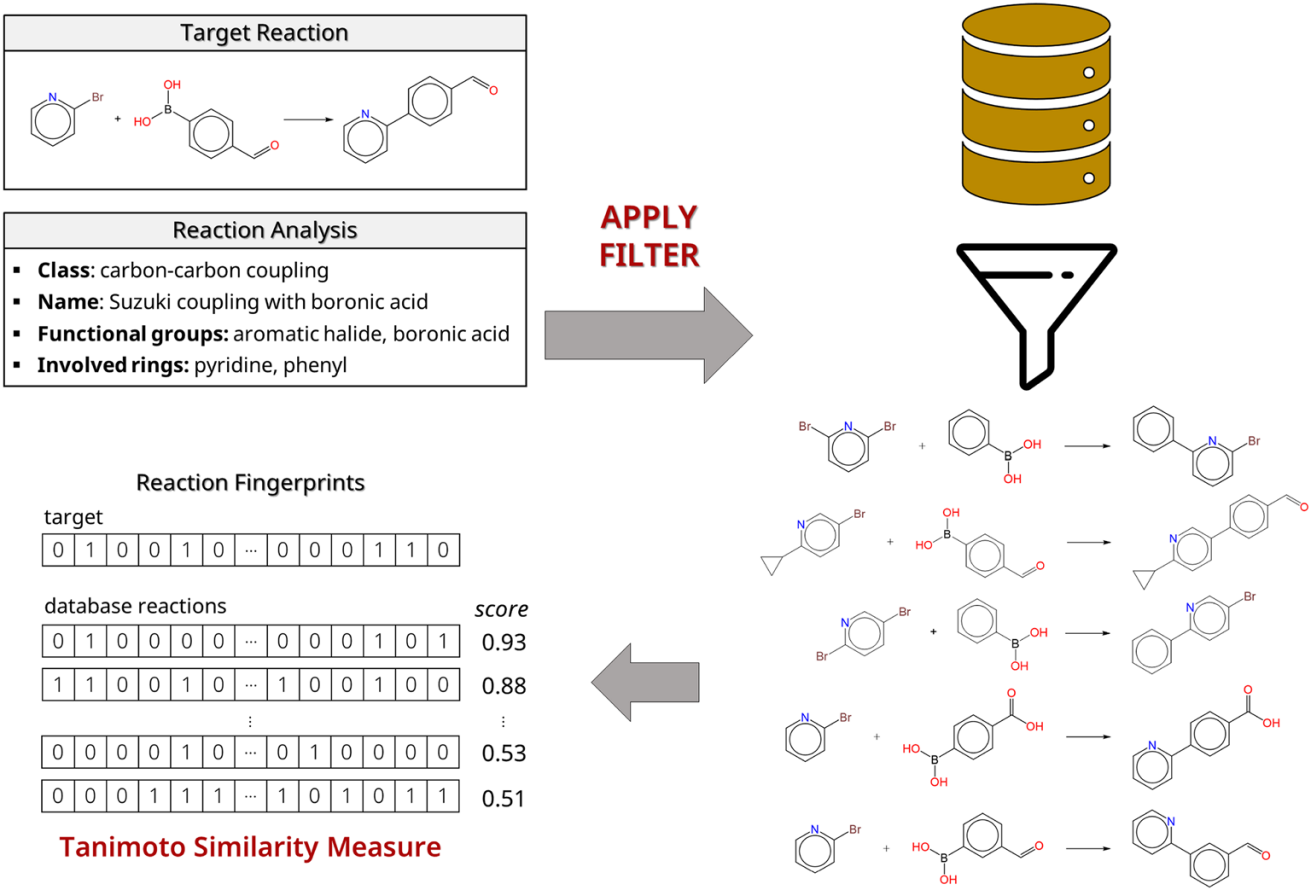
Reaction similarity search. A database is filtered using the reaction analysis of the target reaction. Tanimoto similarity is used to retrieve the most similar reactions

Figure 3 illustrates the workflow of Rxn-INSIGHT’s reaction similarity search. Reaction similarity search is performed on a small subclass of the reaction database. This subclass is defined by reactions that have the same reaction class (*e.g.* C–C coupling), reaction name (*e.g.* Suzuki–Miyaura cross-coupling), the same functional groups in the reaction center, and the same rings that participate in the reaction. The subclass can be further specified by adding the product scaffold or widened by removing the involved rings.

To enable similarity search, reaction fingerprints were constructed for the whole database. Currently, there are four options to represent the reactions using fingerprints that are based on molecular fingerprints. Either 166-bit MACCS keys [48] or 1024-bit extended-connectivity fingerprints with radius 2 (ECFP4; also: Morgan fingerprints) [49] can be used for representing the molecule. The reaction fingerprint is constructed by either adding or concatenating the molecular fingerprints of reactants and products [50]. The default mode uses the concatenated MACCS keys. The reaction similarity is calculated using the Tanimoto distance of the reaction fingerprints [51].

## Results and discussion

### Reaction classification and naming

#### USPTO data evaluation

The Rxn-INSIGHT reaction classification function described in this work is demonstrated on the open-source USPTO patent database [26, 30]. This database contains 1.8 million chemical reactions which are extracted from patents issued between 1976 and 2016. The original data is converted from the XML files provided by Lowe [52] into CSV files containing Reaction SMILES, which are needed as input for Rxn-INSIGHT. A description on the curation of the data is provided in Additional file 1: Section S3) and in the GitHub repository (https://github.com/mrodobbe/Rxn-INSIGHT). The classification and naming of one organic reaction takes between 40 and 100 ms on a laptop with Intel i7 processor, depending on the size of the molecules in the reaction.

**Fig. 4:**
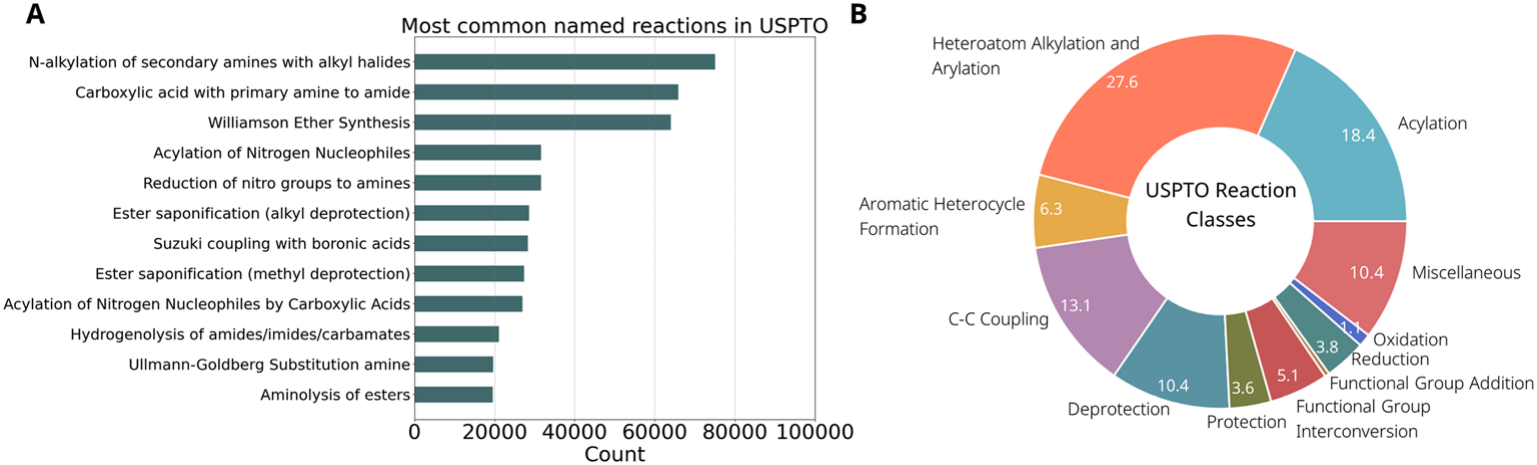
Analysis of 1.8 million reactions in the USPTO database. **A** Distribution of reaction classes. **B** Most common named reactions

We were able to classify 90% of the reactions from USPTO into one of the ten reaction classes, the remainder of the reactions being tagged as “Miscellaneous”. The distribution of the reactions over the reaction classes is given in Fig. 4B. Heteroatom alkylations and arylations are dominant with a share of nearly 30%. In the 2016 analysis by Schneider et al. [30], this reaction class accounted for 27.8% of the reactions. There are several reasons that explain the difference between their results and the results in this work. First of all, the patent data from 2016, which is about 5% of the full database, is not included in the work of Schneider et al*.* [30]. Secondly, the reaction classification is done using the commercial software NameRxn (NextMove) which classifies and names at the same time using SMIRKS identifiers. Since not all reaction motifs are included in the SMIRKS identifiers, some classes might be underrepresented, such as aromatic heterocycle formation. Using the BE-matrix approach, aromatic heterocycle formations are easily recognized by a bond order change of 0.5 or 1.5 in the transformation matrices. In our analysis, this class is the 5th largest, with a share of 6.3% of all reactions.

Using the manually curated SMIRKS patterns, 920,776 reactions (51.2%) in the USPTO database can be named. Figure 4A gives an overview of the most commonly encountered named reactions. The most common reaction type is a rather generally defined N-alkylation of secondary amines with alkyl halides. Another way to find this reaction type is by looking at the class (heteroatom alkylation and arylation) and the functional groups in the reaction center. Then, it can be deduced that in this reaction type a secondary amine reacts with a primary halide, forming a tertiary amine.

#### Benchmark evaluation

We used a labelled subset of 50,000 reactions from the USPTO database to evaluate the naming and classification performance [42]. The dataset counts 50 named reaction types and of each type there are 1000 reactions, all tagged with the reaction name and superclass. Because of the distribution of the reaction classes, this benchmark is a representative dataset for medicinal chemistry tasks, albeit without any aromatic heterocycle formations. The benchmark was constructed with the NameRXN ontology, in which two reactions with the same name are automatically classified in the same reaction class. This is because the NameRXN ontology is purely SMIRKS-based and the reaction name is a subclass of the reaction class. Rxn-INSIGHT handles naming and classifying of a reaction in two independent processes. This is why two reactions with the same name, might be classified into another reaction class. Because different sets of SMIRKS reaction motifs are applied, different levels of detail in naming occur. One such example is the “Methylation” reaction, of which 86.8% of the reactions could be named, albeit with fifteen distinctive methylation motifs. The reaction motifs differ by which methylating agent is used (*e.g.* methyl iodide, dimethyl sulphate, …) or by which atom or functional group is methylated (*e.g.* carbon, hydroxyl, …). The opposite behavior is found in Suzuki-type cross-coupling reactions. In the benchmark dataset, a distinction is made between ‘Chloro Suzuki-type coupling’, ‘Bromo Suzuki-type coupling’, and ‘Bromo Suzuki coupling’, whereas in this work Suzuki-type couplings are named by the other coupling partner (*e.g.* boronic acid).

Table 2 provides an overview of the accuracy of the algorithm per reaction class. Overall, Rxn-INSIGHT was able to correctly assign a name for 95.5% of the reactions and to classify 97.4% of all reactions in the benchmark set. 91.1% of the reactions are classified in the same class as in the NameRXN ontology. However, the lower score for acylations and functional group additions is due to different classification conventions. This work follows the classification convention defined by Roughley and Jordan [29] and is independent of the reaction naming, which explains the different scores for reaction classes and names. A first discrepancy is found for methylation reactions, which are “Functional Group Additions” in the NameRXN ontology, but here classified as “Heteroatom Alkylation and Arylation” or “C–C Coupling”, depending on the alkylated atom. Esterification reactions are the second type that are classified differently, namely as “Protection” in this work and “Acylation” in the NameRXN ontology. According to Roughley and Jordan [29], ester formation by alkylation of a carboxylic acid can be classified both as a “Protection” or “Acylation” depending on the further functionalization of the ester group. A true distinction is only possible by manual evaluation of the full reaction sequence, which is out of scope for both algorithms, so that both reaction classes are valid choices. Because of the possible ambiguity in classification, it is difficult to compare the performance with other classification tools. Rxnfp from Schwaller et al*.* [53] was reported to achieve 98.2% classification accuracy against the NameRXN convention on another USPTO subset when trained on 1000 reaction templates. However, in that work, reactions were classified into 1000 unnamed reaction template classes as opposed to the 10 named reaction classes in this work. Furthermore, the application range of this supervised model is limited to the templates it is trained on, whereas Rxn-INSIGHT can classify reactions without the need for a training set and provide a name by means of rings and functional groups when the template is undefined. Rxn-INSIGHT uses the BE-matrix approach, which relies on atom–atom mapping of a reaction. Despite the high accuracy of RXNMapper [43], the mapping is in some cases inaccurate and leads to a wrong classification, which can partially explain that the classification accuracy is lower than the naming accuracy. Naming a reaction is done via SMIRKS reaction motifs, a process that does not require reaction mapping, so that a wrongly classified reaction can still be named correctly.

**Table 2:** Accuracy of benchmark study for reaction classification and naming

Reaction class	Classification accuracy (%)	Naming accuracy (%)	Count
*Heteroatom alkylation and arylation*	92.8	92.6	14,000
*Acylation*	81.2	97.3	8000
*C–C coupling*	99.8	96.9	5000
*Reduction*	96.5	98.9	4000
*Oxidation*	99.3	97.9	3000
*Protection*	93.9	93.1	1000
*Deprotection*	95.6	95.8	8000
*Functional group addition*	74.4	93.8	5000
*Functional group interconversion*	97.1	99.4	2000
*Total*	91.1	95.5	50,000

### Ring and scaffold detection

Another way to look at a reaction is by evaluating the ring systems in the reaction product. We make a distinction in this work between the rings that are involved in the reaction center, the rings that are formed during reaction, and the scaffold. A ring is said to be involved in the reaction center if the ring itself changes or if a functional group connected to the ring is reacting. Using the information of which rings are involved in the reaction center, a more narrow reaction type can be given. If we revisit the example reaction “Brc1ccccn1.O = Cc1ccc(B(O)O)cc1 >  > O = Cc1ccc(-c2ccccn2)cc1”, then this *Suzuki coupling with boronic acids* is further specified with the information that a pyridine ring and a benzene ring are involved in the reaction center. During the course of this reaction, there are no new rings formed. However, the indication of the formed rings facilitates the naming of aromatic heterocycle formations. In other work, this reaction class was found difficult to name due to the large number of possible heterocycles, but using this ring detection method, it is immediately understandable which functional groups react and which cyclic structure is formed.

#### Enumeration of rings in USPTO reactions

**Fig. 5:**
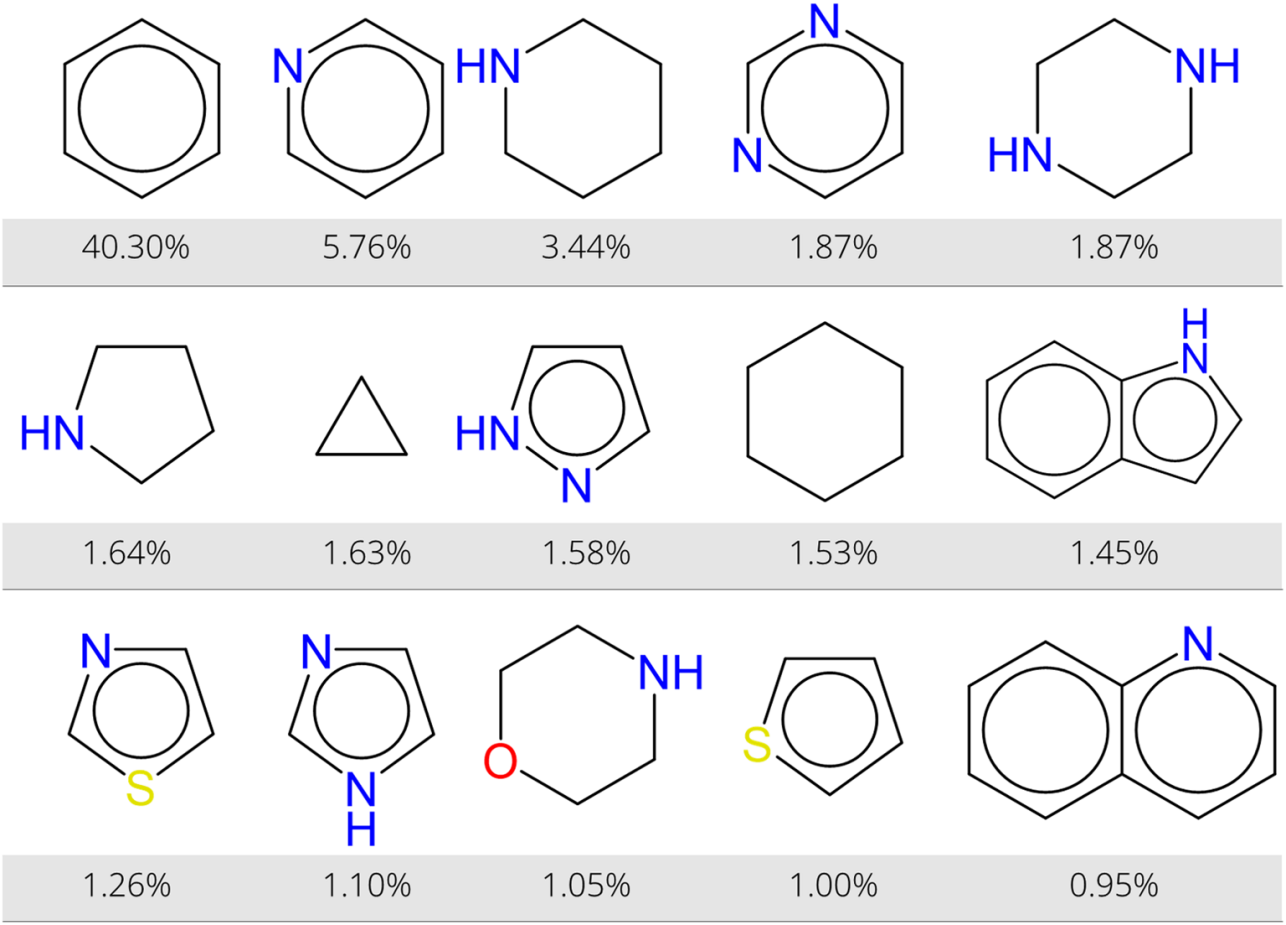
Overview of the most common rings in USPTO products. Benzene, pyridine, piperidine, and pyrimidine cover more than 50% of all rings

The products of all USPTO reactions are analyzed to demonstrate the ring detection feature. We found 965,735 unique products of which only 2.6% is an acyclic compound. These products are not necessarily end points of reaction sequences, but can also be produced reagents or intermediates. We were able to distinguish 18,743 different cyclic structures from all compounds. Of these rings, 5436 are found only once, 1029 are found more than 100 times, and only 201 rings are found in more than 1000 products. Figure 5 shows the fifteen most common rings in USPTO products. As expected a simple phenyl is the most prevalent ring structure. It should be remarked that a polycyclic structure which contains a phenyl (*e.g.* naphthalene) is not included in this count. The other most found cyclic structures are mainly nitrogen-containing heterocycles, such as pyridine, piperidine, pyrimidine, and piperazine.

**Fig. 6:**
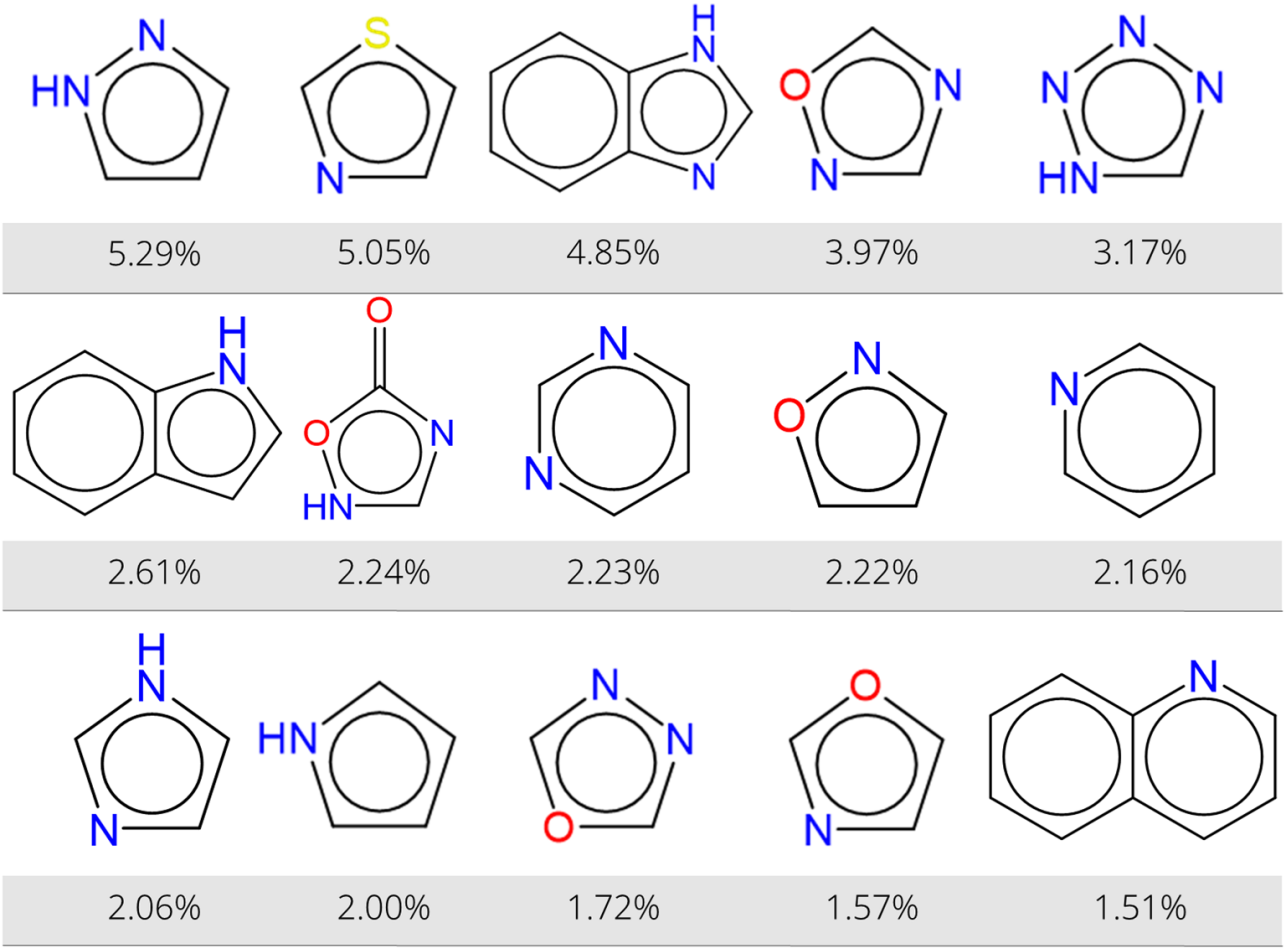
Overview of the most commonly formed aromatic heterocycles in USPTO products. The percentage indicates the relative occurrence among all formed aromatic heterocycles

The formation of aromatic heterocyclic compounds is an important reaction in medicinal chemistry. In Fig. 4b, it was shown that this reaction class accounts for about 6% of all reactions in the USPTO database. However, these reactions are difficult to name using SMIRKS because of the huge amount of possible heterocycles. Indeed, the analysis of 110,000 reactions revealed 3129 different heterocycles formed, of which 105 occur more than 100 times and only 20 heterocycles are formed more than 1000 times. There are 1149 aromatic heterocycles that are formed only in one reaction. Figure 6 presents the fifteen most commonly synthesized aromatic heterocycles. As was also seen in Fig. 5, the majority of these structures are monocyclic and all of the top-15 rings are aza-heterocycles. Among the aromatic heterocyclic compounds that do not contain nitrogen are only 1-benzofuran (0.86%), benzothiophene (0.57%), and thiophene (0.56%) found in more than 500 reactions.

The BE-matrix approach for classification is completely reliant on the atom mapping and will become inaccurate when the atom mapping fails. This means that if the atom mapping for atoms in an aromatic heterocycle is false, a reaction can be labeled incorrectly as an aromatic heterocycle formation. Similarly, if an aromatic reactant is missing because of incorrect stoichiometry but the aromatic ring is given in the product, then the reaction can be falsely classified as an aromatic heterocycle formation. Where the NameRxn method underestimated aromatic heterocycle formations [30], our work is potentially slightly overestimating the number of aromatic heterocycle formations.

#### Extraction of molecular scaffolds in USPTO reactions

**Fig. 7:**
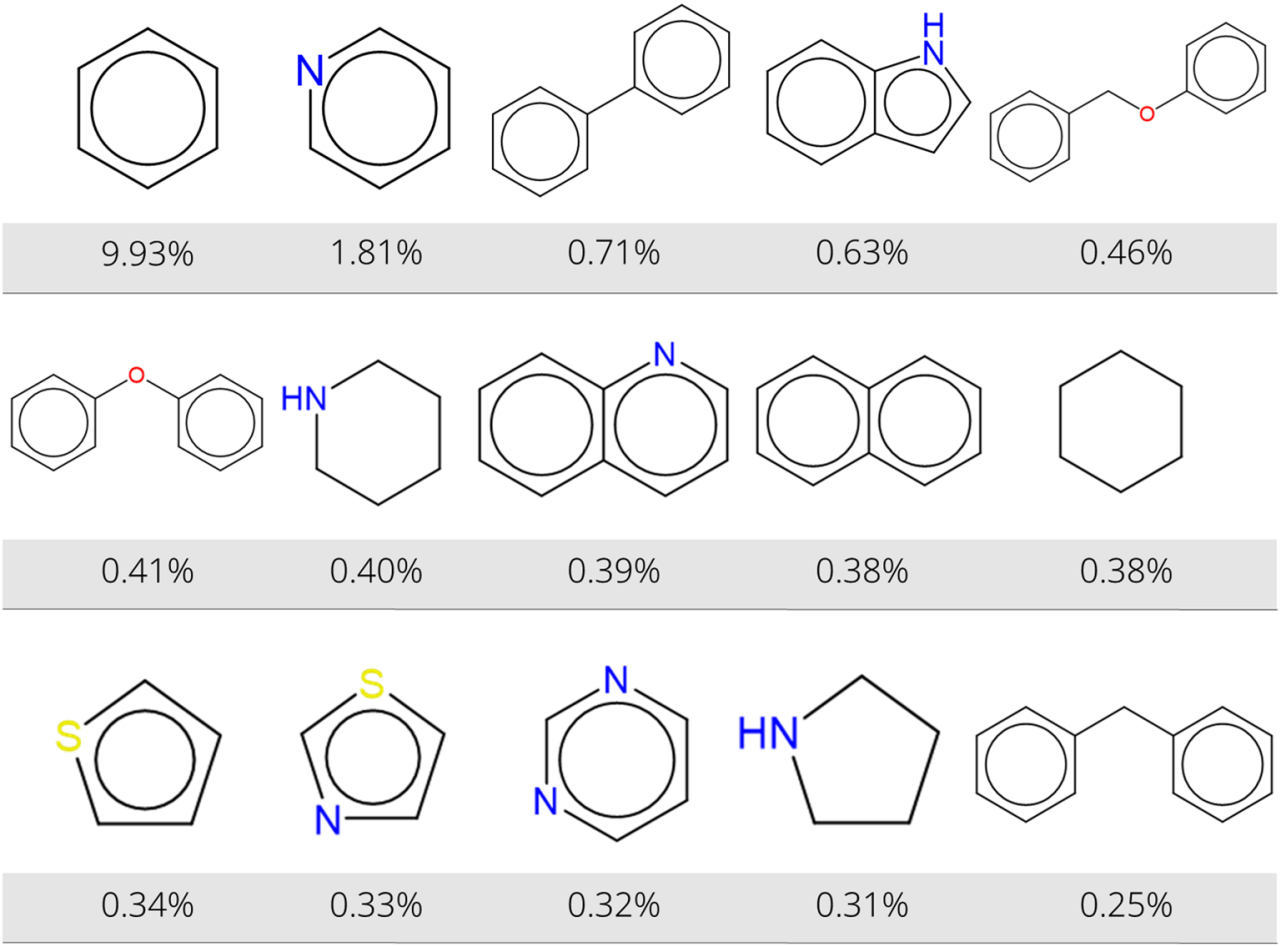
Overview of the most common Bemis-Murcko scaffolds in USPTO products. The percentage indicates the relative occurrence among all scaffolds

We have tested the Rxn-INSIGHT functionality to extract the Bemis-Murcko (BM) scaffold [47] on the major product in all reactions from the USPTO database. There are 251,116 unique BM scaffolds of which 110,365 occur just once, and only 1275 scaffolds are found in the products of more than 100 reactions. The fifteen most common BM scaffolds in USPTO products are illustrated in Fig. 7. Similarly to the most common ring structures, benzene is the most common scaffold, found in 9.9% of all reaction products. Among the other most prevalent scaffolds are many monocyclic structures, such as pyridine (1.8%), piperidine (0.40%), cyclohexane (0.38%), thiophene (0.34%), and thiazole (0.33%). Among the polycyclic scaffolds are several linked phenyl rings, such as biphenyl (0.71%), phenoxymethylbenzene (0.46%), phenoxybenzene (0.41%), and diphenylmethane (0.25%). The prevalence of these phenyl-based scaffolds is in agreement with the large number of phenyl rings seen in the database. Additionally, the large amount of biphenyls and phenyl ethers can explain the popularity of aryl-aryl coupling reactions and of the Williamson ether synthesis. The imbalance in the USPTO database for medicinal chemistry applications is shown by comparing the BM scaffolds in USPTO with the BM scaffolds of approved small molecule drugs. A selection of 2434 drugs, taken from ChEMBL [54], is analyzed. It is found that they are composed of 1243 different BM scaffolds. These 1243 scaffolds make up 72.8% of all scaffolds in USPTO products. In total, 82.5% of the BM scaffolds of approved drugs are covered by USPTO product scaffolds. The complete list of scaffolds in USPTO reactions and in approved drugs are found in Additional file 1.

### Similarity search

**Fig. 8:**
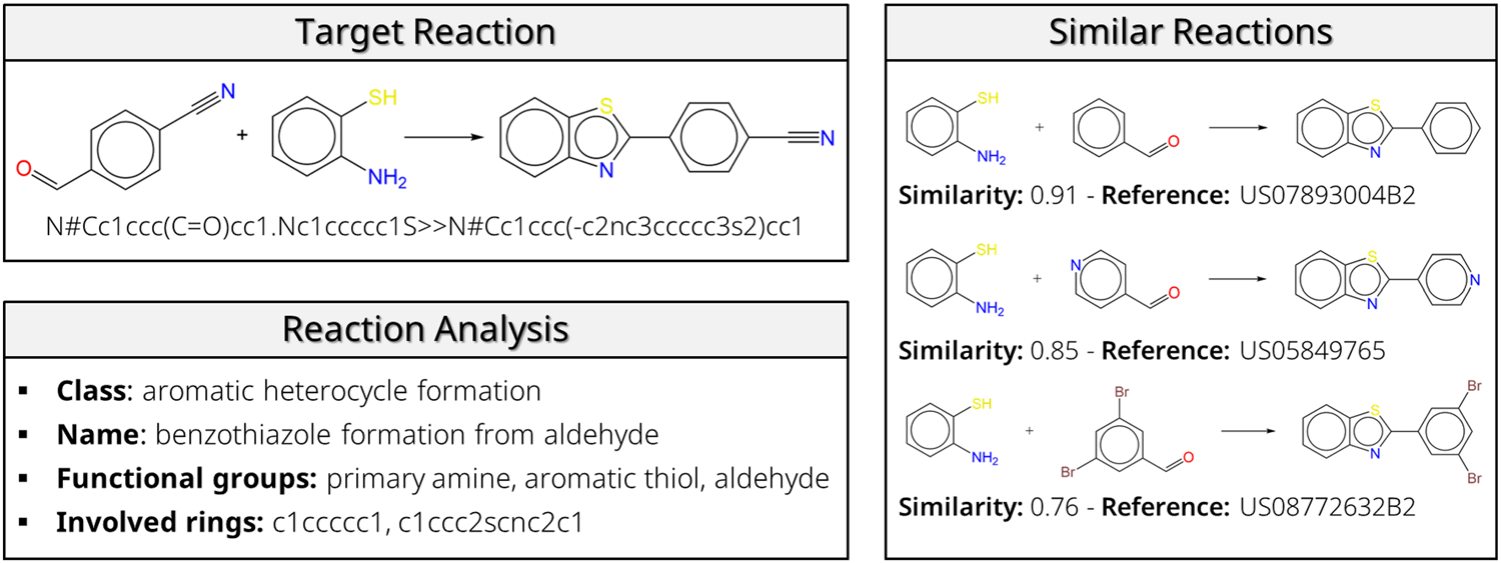
Reaction similarity search. Similar reactions are searched by considering a subset of the database that contains reactions with the same reaction class and involved functional groups. Tanimoto distance of reaction fingerprints is used for similarity measurement

A convenient way to retrieve reaction conditions for a new reaction is based on similarity. Since the number of published reactions and associated conditions is very high, screening the literature quickly turns into a time-consuming task. In this work, any database with chemical reactions and conditions can be screened. There are three different screening options. The first method looks at reactions with the exact same reaction class, name, and involved functional groups and rings. This is a very tight search that is performed in less than a second. Because of the large diversity of rings, the request can be too narrow, especially for less common reactions. The second method offers a solution to this potential problem by broadening the search. Only the reaction class and the participating functional groups are considered in this mode. An example is shown in Fig. 8. The target reaction is labeled as a “benzothiazole formation from aldehyde”, in which a primary amine, aromatic thiol, and an aldehyde react. When the first search method is used, only reactions are shown in which both reactants have phenyl groups. Thus, the second similar reaction in Fig. 8 is only returned when a broadened search is requested, since one of the reactants contains a pyridine ring. The third way to search is by screening the complete database. For the 1.8 million USPTO reactions, it takes about 7 min on a laptop with Intel i7 processor to measure the Tanimoto distances with all other reactions. This feature is especially handy when using smaller, more task-specific reaction datasets. In all modes, it is possible to set a threshold value for the similarity search. This threshold value is dependent on the fingerprint that is used. When the difference MACCS fingerprints are used, the similarity of the target reaction with the most similar reaction is 0.914, compared to 0.906 with concatenated MACCS fingerprints. The similarity values for ECFP4 (“Morgan fingerprints”) are lower, respectively 0.625 and 0.694 for the concatenated and difference reaction fingerprints. Therefore, a higher threshold value is suggested when using MACCS reaction fingerprints.

### Reaction condition suggestion

**Fig. 9:**
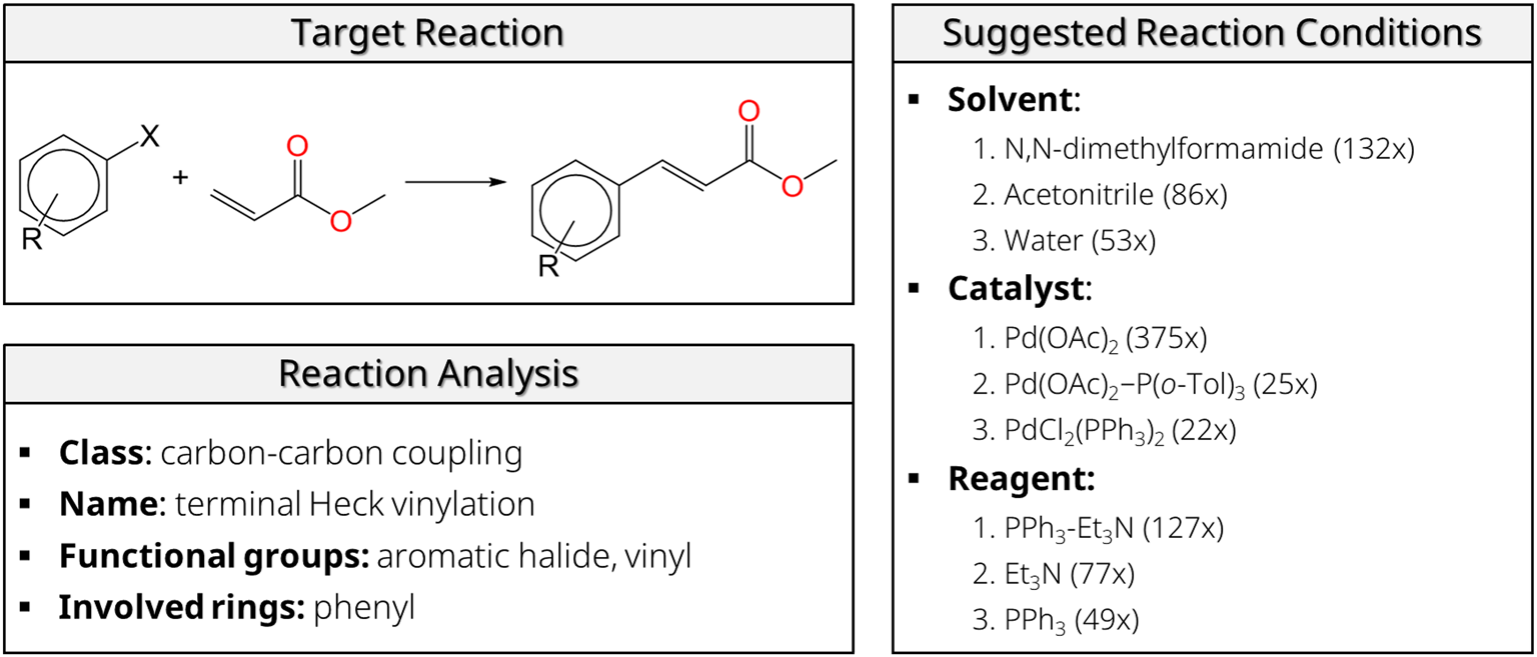
Suggestion of reaction conditions for Heck cross-coupling reactions of aromatic halides and methyl acrylate. Most frequently used solvent, catalyst, and reagent are given with the frequency of occurrence. X = I, Br. R = H, 2-NO_2_, 3-NO_2_, 2-COCH_3_, 4-COCH_3_, 4-Cl, 4-CN

Reaction condition suggestion is a by-product from the reaction classification, naming, and similarity tasks in Rxn-INSIGHT. Indeed, the subset of reactions in the database with the same name, class, and functional groups gives an indication of which solvent, catalyst, and reagent can be used for the new target reaction. As an illustrative case study, we demonstrate the condition suggestion task on 9 Heck cross-coupling reactions between substituted iodo- and bromobenzenes and methyl acrylate (see Fig. 9), from Parker et al*.* [55]. All reactions are recognized by Rxn-INSIGHT as carbon–carbon couplings, more specifically as terminal Heck vinylations with an aromatic halide. Hence, in the similarity search, the same subset of approximately 2000 Heck-type reactions is selected from the USPTO dataset and the same reaction conditions suggestion is made for all 9 target reactions. Figure 9 shows the top-3 solvent, reagent, and catalyst suggestions from Rxn-INSIGHT for these Heck reactions with the frequency of occurrence. It is well-documented that the suggested dipolar aprotic solvents (DMF, acetonitrile) and water are very commonly used in Heck cross-coupling reactions [56], but Rxn-INSIGHT only screens the database and does not take into account the solubility of the involved species in the solvent. Therefore, suggested solvents might not be effective in every variant of the reaction if the reactivity is heavily dependent on the solvent. From this subset, Rxn-INSIGHT also suggests to use Pd(OAc)_2_ as a catalyst, and triethylamine and triphenylphosphine as reagents, which are respectively used as base and as ligand.

## Conclusion

In this work, we have introduced a software tool that is able to rapidly analyze and screen large reaction databases. A rule-based classification algorithm was constructed that sorts a reaction in a certain reaction class (*e.g.* functional group interconversion) using a bond-electron matrix approach. It is found that patterns in a mathematical representation of reaction centers can be linked to a human-developed reaction class without the need for parametrized models. We used the algorithm to analyze a public chemical reaction database of 1.8 million unlabeled reactions and were able to classify 90% of the reactions and give a specific name to more than 50% of the reactions with a set of 527 reaction motifs. On a benchmark dataset of 50,000 reactions, labeled with reaction classes and names, a classification accuracy of more than 90% and a naming accuracy over 95% was found. The choice of where to classify some reaction types is often debatable and not always straightforward, which can lead to inconsistencies between different classification tools. Since the matrix approach is fully reliant on the atom–atom mapping of a reaction, the accuracy is limited by the accuracy of the mapping method. The tool also allows to analyze all the compounds in the reactions, with extraction of functional groups, rings, and molecular scaffolds. The ability to classify and name a reaction with the extraction of functional groups and rings in the reaction center divides the reaction database in smaller subsets. These subsets are then used to retrieve similar reactions and their synthesis conditions using the Tanimoto distance of reaction fingerprints. Based on the reaction similarity and the occurrence, a solvent, catalyst, and reagent can be suggested to the user as a side application. This way of classifying reaction databases to look up new reaction conditions mimics a chemist’s way of screening literature to find reaction conditions of a new chemical reaction. Our model is publicly available on GitHub (https://github.com/mrodobbe/Rxn-INSIGHT) and can be applied to any reaction database.

## Scientific contribution

This tool enhances computer-aided organic synthesis by efficiently screening large chemical databases and accurately classifying and naming chemical reactions. Its ability to divide databases into subsets with similar reactions facilitates the rapid and reliable analysis of suitable conditions, which is still a missing link in computer-aided synthesis planning software. The tool’s high speed and explainability, demonstrated with examples from literature, make it a valuable addition for integration with other software tools, contributing to the overall advancement of synthesis planning.

### Supplementary Information

**Additional file 1.** **Note S1**. Code availability statement. **Note S2**. Data availability statement. **Note S3**. Curation of the USPTO database. **Note S4**. Reaction naming. **Note S5**. Functional group data.

**Additional file 2.** Spreadsheet with SMIRKS identifiers for named reaction patterns.

## Data Availability

The source code is available in the GitHub repository: https://github.com/mrodobbe/Rxn-INSIGHT. The analyzed USPTO database is available on Zenodo: https://doi.org/10.5281/zenodo.10171745.

## References

[1] Blakemore DC, Castro L, Churcher I, Rees DC, Thomas AW, Wilson DM, Wood A (2018). Organic synthesis provides opportunities to transform drug discovery. Nat Chem.

[2] Corey EJ (1967). General methods for the construction of complex molecules. Pure Appl Chem.

[3] Cook A, Johnson AP, Law J, Mirzazadeh M, Ravitz O, Simon A (2011). Computer-aided synthesis design: 40 years on. WIREs Comput Mol Sci.

[4] Todd MH (2005). Computer-aided organic synthesis. Chem Soc Rev.

[5] Ihlenfeldt W-D, Gasteiger J (1996). Computer-assisted planning of organic syntheses: the second generation of programs. Angew Chem Int Ed Engl.

[6] Coley CW, Green WH, Jensen KF (2018). Machine learning in computer-aided synthesis planning. Acc Chem Res.

[7] Thakkar A, Johansson S, Jorner K, Buttar D, Reymond J-L, Engkvist O (2021). Artificial intelligence and automation in computer aided synthesis planning. React Chem Eng.

[8] Dobbelaere MR, Plehiers PP, Van de Vijver R, Stevens CV, Van Geem KM (2021). Machine learning in chemical engineering: strengths, weaknesses, opportunities, and threats. Engineering.

[9] Szymkuc S, Gajewska EP, Klucznik T, Molga K, Dittwald P, Startek M, Bajczyk M, Grzybowski BA (2016). Computer-assisted synthetic planning: the end of the beginning. Angew Chem Int Ed Engl.

[10] Segler MHS, Preuss M, Waller MP (2018). Planning chemical syntheses with deep neural networks and symbolic AI. Nature.

[11] Genheden S, Thakkar A, Chadimova V, Reymond JL, Engkvist O, Bjerrum E (2020). AiZynthFinder: a fast, robust and flexible open-source software for retrosynthetic planning. J Cheminform.

[12] Schwaller P, Petraglia R, Zullo V, Nair VH, Haeuselmann RA, Pisoni R, Bekas C, Iuliano A, Laino T (2020). Predicting retrosynthetic pathways using transformer-based models and a hyper-graph exploration strategy. Chem Sci.

[13] Hendrickson JB (1990). Organic synthesis in the age of computers. Angew Chem Int Ed Engl.

[14] Ayers PW, Anderson JSM, Bartolotti LJ (2005). Perturbative perspectives on the chemical reaction prediction problem. Int J Quantum Chem.

[15] Warr WA (2014). A short review of chemical reaction database systems, computer-aided synthesis design, reaction prediction and synthetic feasibility. Mol Inf.

[16] Fooshee D, Mood A, Gutman E, Tavakoli M, Urban G, Liu F, Huynh N, Van Vranken D, Baldi P (2018). Deep learning for chemical reaction prediction. Mol Syst Des Eng.

[17] Schwaller P, Laino T, Gaudin T, Bolgar P, Hunter CA, Bekas C, Lee AA (2019). Molecular transformer: a model for uncertainty-calibrated chemical reaction prediction. ACS Cent Sci.

[18] Venkatasubramanian V, Mann V (2022). Artificial intelligence in reaction prediction and chemical synthesis. Curr Opin Chem Eng.

[19] Coley CW, Thomas DA, Lummiss JAM, Jaworski JN, Breen CP, Schultz V, Hart T, Fishman JS, Rogers L, Gao H (2019). A robotic platform for flow synthesis of organic compounds informed by AI planning. Science.

[20] Shields BJ, Stevens J, Li J, Parasram M, Damani F, Alvarado JIM, Janey JM, Adams RP, Doyle AG (2021). Bayesian reaction optimization as a tool for chemical synthesis. Nature.

[21] Ureel Y, Dobbelaere MR, Ouyang Y, De Ras K, Sabbe MK, Marin GB, Van Geem KM (2023). Active machine learning for chemical engineers: a bright future lies ahead!. Engineering.

[22] Beker W, Roszak R, Wolos A, Angello NH, Rathore V, Burke MD, Grzybowski BA (2022). Machine learning may sometimes simply capture literature popularity trends: a case study of heterocyclic Suzuki-Miyaura coupling. J Am Chem Soc.

[23] Marcou G, de Sousa JA, Latino DA, de Luca A, Horvath D, Rietsch V, Varnek A (2015). Expert system for predicting reaction conditions: the Michael reaction case. J Chem Inf Model.

[24] Gao H, Struble TJ, Coley CW, Wang Y, Green WH, Jensen KF (2018). Using machine learning to predict suitable conditions for organic reactions. ACS Cent Sci.

[25] Mercado R, Kearnes SM, Coley CW (2023). Data sharing in chemistry: lessons learned and a case for mandating structured reaction data. J Chem Inf Model.

[26] Lowe DM (2012). Extraction of chemical structures and reactions from the literature.

[27] Kearnes SM, Maser MR, Wleklinski M, Kast A, Doyle AG, Dreher SD, Hawkins JM, Jensen KF, Coley CW (2021). The open reaction database. J Am Chem Soc.

[28] Carey JS, Laffan D, Thomson C, Williams MT (2006). Analysis of the reactions used for the preparation of drug candidate molecules. Org Biomol Chem.

[29] Roughley SD, Jordan AM (2011). The medicinal chemist’s toolbox: an analysis of reactions used in the pursuit of drug candidates. J Med Chem.

[30] Schneider N, Lowe DM, Sayle RA, Tarselli MA, Landrum GA (2016). Big data from pharmaceutical patents: a computational analysis of medicinal chemists’ bread and butter. J Med Chem.

[31] Bostrom J, Brown DG, Young RJ, Keseru GM (2018). Expanding the medicinal chemistry synthetic toolbox. Nat Rev Drug Discov.

[32] Weininger D SMIRKS—a reaction transform language. Daylight Chemical Information Systems Inc. https://www.daylight.com/dayhtml/doc/theory/theory.smirks.html, Accessed 23 Jan 2023

[33] Hartenfeller M, Eberle M, Meier P, Nieto-Oberhuber C, Altmann KH, Schneider G, Jacoby E, Renner S (2011). A collection of robust organic synthesis reactions for in silico molecule design. J Chem Inf Model.

[34] Avramova S, Kochev N, Angelov P (2018). RetroTransformDB: a dataset of generic transforms for retrosynthetic analysis. Data.

[35] Plehiers PP, Marin GB, Stevens CV, Van Geem KM (2018). Automated reaction database and reaction network analysis: extraction of reaction templates using cheminformatics. J Cheminform.

[36] Coley CW, Green WH, Jensen KF (2019). RDChiral: an RDKit wrapper for handling stereochemistry in retrosynthetic template extraction and application. J Chem Inf Model.

[37] Visini R, Arus-Pous J, Awale M, Reymond JL (2017). Virtual exploration of the ring systems chemical universe. J Chem Inf Model.

[38] Ertl P (2022). Magic rings: navigation in the ring chemical space guided by the bioactive rings. J Chem Inf Model.

[39] Ertl P (2017). An algorithm to identify functional groups in organic molecules. J Cheminform.

[40] CAS reactions. American Chemical Society. https://www.cas.org/cas-data. Accessed 11 July 2023

[41] Landrum GA RDKit: open-source cheminformatics. (Version Q1 2023) https://www.rdkit.org/. Accessed 9 May 2023

[42] Schwaller P, Hoover B, Reymond J-L, Strobelt H, Laino T (2021). Extraction of organic chemistry grammar from unsupervised learning of chemical reactions. Sci Adv.

[43] Lin A, Dyubankova N, Madzhidov TI, Nugmanov RI, Verhoeven J, Gimadiev TR, Afonina VA, Ibragimova Z, Rakhimbekova A, Sidorov P (2022). Atom-to-atom mapping: a benchmarking study of popular mapping algorithms and consensus strategies. Mol Inform.

[44] Dugundji J, Ugi I (1973) An algebraic model of constitutional chemistry as a basis for chemical computer programs. In: Computers in Chemistry. Springer Berlin Heidelberg, Berlin, Heidelberg

[45] Spialter L (1963). The atom connectivity matrix (ACM) and its characteristic polynomial (ACMCP): a new computer-oriented chemical nomenclature. J Am Chem Soc.

[46] Weininger D SMARTS—a language for describing molecular patterns. Daylight Chemical Information Systems Inc. https://www.daylight.com/dayhtml/doc/theory/theory.smarts.html. Accessed 23 Jan 2023

[47] Bemis GW, Murcko MA (1996). The properties of known drugs. 1. Molecular frameworks. J Med Chem.

[48] Durant JL, Leland BA, Henry DR, Nourse JG (2002). Reoptimization of MDL keys for use in drug discovery. J Chem Inf Comput Sci.

[49] Rogers D, Hahn M (2010). Extended-connectivity fingerprints. J Chem Inf Model.

[50] Schneider N, Lowe DM, Sayle RA, Landrum GA (2015). Development of a novel fingerprint for chemical reactions and its application to large-scale reaction classification and similarity. J Chem Inf Model.

[51] Bajusz D, Rácz A, Héberger K (2015). Why is Tanimoto index an appropriate choice for fingerprint-based similarity calculations?. J Cheminf.

[52] Lowe D (2017) Chemical reactions from US patents (1976–Sep 2016). *figshare*

[53] Schwaller P, Probst D, Vaucher AC, Nair VH, Kreutter D, Laino T, Reymond J-L (2021). Mapping the space of chemical reactions using attention-based neural networks. Nat Mach Intell.

[54] Gaulton A, Hersey A, Nowotka M, Bento AP, Chambers J, Mendez D, Mutowo P, Atkinson F, Bellis LJ, Cibrián-Uhalte E (2016). The ChEMBL database in 2017. Nucleic Acids Res.

[55] Parker HL, Sherwood J, Hunt AJ, Clark JH (2014). Cyclic carbonates as green alternative solvents for the heck reaction. ACS Sustain Chem Eng.

[56] Beletskaya IP, Cheprakov AV (2000). The heck reaction as a sharpening stone of palladium catalysis. Chem Rev.

