# Supplementary material for: Rxn-INSIGHT: fast chemical reaction analysis using bond-electron matrices
Source: J Cheminform. 2024 Mar 29;16:37. doi: 10.1186/s13321-024-00834-z (PMC10980627; doi:10.1186/s13321-024-00834-z)
Supplement: Supplementary file 1 — Additional file 1. Note S1. Code availability statement. Note S2. Data availability statement. Note S3. Curation of the USPTO database. Note S4. Reaction naming. Note S5. Functional group data. [file 13321_2024_834_MOESM1_ESM.docx]

**Rxn-INSIGHT: Fast Chemical Reaction Analysis Using Bond-Electron Matrices**

Maarten R. Dobbelaere^1^, István Lengyel^1,2^, Christian V. Stevens^3^, Kevin M. Van Geem^1,*^

^1^Laboratory for Chemical Technology, Department of Materials, Textiles and Chemical Engineering, Faculty of Engineering and Architecture, Ghent University, Technologiepark 125, 9052 Gent, Belgium

^2^ChemInsights LLC, Dover DE 19901, United States of America

^3^SynBioC Research Group, Department of Green Chemistry and Technology, Faculty of Bioscience Engineering, Ghent University, Coupure Links 653, 9000 Gent, Belgium

^*^ Corresponding author: [Kevin.VanGeem@UGent.be](mailto:Kevin.VanGeem@UGent.be), Technologiepark 125, 9052 Gent, Belgium

**SUPPORTING INFORMATION**

# Code Availability

The complete source code is provided as open-source software under MIT license on the GitHub repository: <https://github.com/mrodobbe/rxn-insight>.

The various functions of Rxn-INSIGHT can be tested out in a ready-to-run demo Jupyter notebook demo.ipynb.

# Data Availability

The analyzed USPTO database can be downloaded from Zenodo (<https://doi.org/10.5281/zenodo.10171745>) under the name uspto_rxn_insight.gzip. This gzip file contains a very large Pandas DataFrame that can be loaded via pd.read_parquet(‘uspto_rxn_insight.gzip’). Because of the large size of the data, PyArrow version $\leq$13.0 must be used.

To use parquet in Pandas, install PyArrow and fastparquet using pip:

pip install pyarrow==13.0

pip install fastparquet

# USPTO Data Curation

The USPTO database is downloaded from <https://figshare.com/articles/dataset/Chemical_reactions_from_US_patents_1976-Sep2016_/5104873>. The initial data format is XML and lists all reactants, products, solvents, catalysts, and reagents separately. The Reaction SMILES were given with original mapping. All data is converted into CSV files per grant year (*e.g.,* “1976_USPTO.csv” contains all reactions reported in 1976) in which the reactants, products, an unmapped Reaction SMILES, the solvents, reagents, catalysts, and the reference number are given. The reaction classification and naming is done using the method described in the manuscript with an unmapped Reaction SMILES as sole input. To make the study as complete as possible, the USPTO database is kept in its entirety as originally submitted on FigShare. The script used for converting the XML files is found on <https://github.com/mrodobbe/Rxn-INSIGHT/blob/master/convert_xml.py>.

# Reaction Naming

527 SMIRKS are defined for reaction naming and can be found in the spreadsheet smirks.xlsx, found in supporting information. SMIRKS that are noted with {} come from Hartenfeller *et al.* [[1](#_ENREF_1)]. The SMIRKS are also found in JSON-format in the GitHub repository: <https://github.com/mrodobbe/Rxn-INSIGHT/blob/master/json/smirks.json>. The JSON file can be read as follows:

pd.read_json(smirks.json, orient='records', lines=True)

# Functional Groups

Functional groups are detected by matching the molecule with 107 SMARTS patterns. The SMARTS are found in JSON-format in the GitHub repository: <https://github.com/mrodobbe/Rxn-INSIGHT/blob/master/json/functional_groups.json>. The JSON file can be read as follows:

pd.read_json(functional_groups.json, orient='records', lines=True)

# References

1. Hartenfeller, M.; Eberle, M.; Meier, P.; Nieto-Oberhuber, C.; Altmann, K.H.; Schneider, G.; Jacoby, E.; Renner, S. A collection of robust organic synthesis reactions for in silico molecule design*.* *J Chem Inf Model* **2011,** *51*, 3093-8.
